# Epigenetic landscape change analysis during human EMT sheds light on a key EMT mediator TRIM29

**DOI:** 10.18632/oncotarget.21681

**Published:** 2017-10-09

**Authors:** Sung Kyung Choi, Kurinji Pandiyan, Jung Woo Eun, Xiaojing Yang, Seong Hwi Hong, Suk Woo Nam, Peter A. Jones, Gangning Liang, Jueng Soo You

**Affiliations:** ^1^ Department of Biochemistry, School of Medicine, Konkuk University, Seoul, Korea; ^2^ Departments of Urology, Keck School of Medicine, University of Southern California, Los Angeles, CA, USA; ^3^ Department of Oncology, Sidney Kimmel Comprehensive Cancer Center, Johns Hopkins School of Medicine, Baltimore, MD, USA; ^4^ Department of Pathology, College of Medicine, The Catholic University, Seoul, Korea; ^5^ Van Andel Research Institute, Grand Rapids, MI, USA; ^6^ Research Institute of Medical Science, KonKuk University School of Medicine, Seoul, Korea

**Keywords:** epigenetic landscape, EMT, DNA methylation, chromatin accessibility, TRIM29

## Abstract

Epithelial to mesenchymal transition (EMT) is a key trans-differentiation process, which plays a critical role in physiology and pathology. Although gene expression changes in EMT have been scrutinized, study of epigenome is in its infancy. To understand epigenetic changes during TWIST-driven EMT, we used the AcceSssIble assay to study DNA methylation and chromatin accessibility in human mammary epithelial cells (HMECs). The DNA methylation changes were found to have functional significance in EMT – i.e. methylated genes were enriched for E-box motifs that can be recognized by TWIST, at the promoters suggesting a potential targeting phenomenon, whereas the demethylated regions were enriched for pro-metastatic genes, supporting the role of EMT in metastasis. TWIST-induced EMT triggers alterations in chromatin accessibility both independent of and dependent on DNA methylation changes, primarily resulting in closed chromatin conformation. By overlapping the genes, whose chromatin structure is changed during early EMT and a known “core EMT signature”, we identified 18 driver candidate genes during EMT, 14 upregulated and 4 downregulated genes with corresponding chromatin structure changes. Among 18 genes, we focused on TRIM29 as a novel marker of EMT. Although loss of TRIM29 is insufficient to suppress CDH, it is enough to induce CDH2 and VIM. Gene functional annotation analysis shows the involvement of TRIM29 in epidermal development, cell differentiation and cell migration. Taken together, our results provide a robust snapshot of chromatin state during human EMT and identify TRIM29 as a core mediator of EMT.

## INTRODUCTION

Epithelial to mesenchymal transition (EMT) allows the trans-differentiation of epithelial and endothelial cell types to mesenchymal cells and plays important roles in normal development and disease progression [1, 2]. EMT and the converse process of mesenchymal to epithelial transition (MET) are reversible; several rounds of EMT and MET are required for the proper differentiation of cells and organs during development [2]. Transcriptional repressors, such as SNAI1 (Snail), SNAI2 (Slug) and TWIST are key players in the process and have been shown to repress pro-epithelial/adhesion proteins, working in concert to induce the transformation of locally restricted epithelial cells into a migratory phenotype resulting in dramatic tissue re-organization [3]. CDH1 (E-Cadherin) as a component of adherens junctions, is the best-studied mediator of adhesion in epithelial cells [4]. Although EMT was initially recognized as a developmental process, it has been shown to occur in adults during wound healing [5]. It is also well established that tumors hijack this mechanism during metastasis, for invasion, intravasation, movement through circulation, and extravasation [6]. Owing to the importance of EMT in tumorigenesis and wound healing, a deep understanding of the process and its players is required to manipulate the system for therapeutic purposes.

Gene expression is regulated by multiple layers of epigenetic control, including DNA methylation, histone modifications, and nucleosome positioning [7, 8]. While the former two have been extensively studied, the importance of nucleosome positioning and chromatin accessibility in development and disease is now surfacing [9, 10], after the realization that nucleosome deposition precedes and is essential for the DNA methylation and histone modifications that accompany such processes [11]. Further, the importance of nucleosome depletion/chromatin accessibility at transcription start sites (TSSs) and at enhancers in enabling gene expression has been recently established [12, 13].

Current methods for studying chromatin accessibility or nucleosome footprints involve the coupling of next generation sequencing with chromatin immunoprecipitation (ChIP) for histone H3, with nuclease digestion [14, 15] or Formaldehyde-Assisted Isolation of Regulatory Elements (FAIRE) [16]. Recently, others and we have developed methylation-sensitive single molecule analyses M-SPA (Methyltransferase-based Single Promoter Analysis) and NOME (Nucleosome Occupancy and DNA Methylome)-seq to study nucleosome positioning [11, 17–19]. These methods exploit the ability of CpG (M.SssI) or GpC (M.CvipI) methyltransferases to methylate CpG or GpC dinucleotides respectively, in native chromatin that is devoid of nucleosomes and tightly bound with transcription factors. More recently, we have developed a genome-wide extension for investigators seeking a global view of chromatin accessibility and DNA methylation at a modest-resolution and in a cost-effective manner [20]. This method, named as AcceSssIble, involves treatment of chromatin with the commercially available CpG methyltransferase SssI, and subsequent assay on the widely used Illumina HumanMethylation450 platform [21] along with a no-enzyme control. Using this method, we have identified that functional DNA demethylation is accompanied by chromatin accessibility [20].

Efforts have been made to determine the underlying mechanisms of EMT and to demonstrate the epigenetic reprogramming involved in the process [22, 23]. Few studies have carefully characterized the changes in the epigenetic landscape that allows for this dramatic transformation in cell fate. One of the first studies that studied these changes globally was conducted by the Feinberg group [24]. In this study, mouse epithelial cells were treated with transforming growth factor-beta (TGF-beta), a known inducer of EMT, and DNA methylation changes were characterized using global methylation arrays. It was observed that DNA methylation was largely unchanged during the process. The study attributed a Lysine Specific Demethylase (LSD1)-driven global increase in euchromatin, including a decrease in the repressive H3K9me2 mark and an increase in the activating H3K4me3 mark in specific domains across the genome, as key epigenetic changes during the EMT process [24]. While the study provides interesting preliminary insights in the epigenomics of EMT, much remains to be discovered. It has been shown that there are numerous differences mark between human and mouse development and hence, it is crucial to extend the study to a human model of EMT [25, 26]. Additionally, since this study used only one model system to study EMT, the question remains whether EMT induction by different methods results in the same epigenomic changes.

To address these open questions regarding epigenetic changes mediating EMT, we explored EMT induced by the transcriptional repressor TWIST in the human mammary epithelial system. We assessed DNA methylation and chromatin accessibility in a rapid and cost effective manner using AcceSssIble in these cells, at several time points throughout the course of EMT. Next, we identified 18 driver candidate genes during EMT considering change of chromatin structure and mRNA expression. Among EMT driver candidates, knockdown of TRIM29 in HMECs followed by transcriptome analysis revealed that TRIM29 is a key mediator of EMT and other cellular processes. Altogether, our data provides two important epigenetic maps of DNA methylation and chromatin accessibility during EMT in human system and identifies 18 high probability markers of EMT including the novel mediator TRIM29.

## RESULTS

### TWIST-induced EMT is accompanied by changes in DNA methylation

To survey the epigenetic changes that occur during EMT, we employed a TWIST-driven EMT model system that has been previously described [27]. We observed that the treated cells displayed mesenchymal properties that are characteristic of EMT, i.e. loss of cell-cell adhesion and change in morphology from cobblestone shaped cells to spindle shaped cells (Supplementary Figure 1A). To confirm the transition at the molecular level, we performed quantitative real-time RT-PCR analysis for known mesenchymal markers in EMT including TWIST, CDH2 (N-Cadherin), SNAI1 (Snail) and VIM (Vimentin), as well as the epithelial marker CDH1 (E-Cadherin). TWIST-driven EMT resulted in downregulation of CDH1 and upregulation of CDH2 and VIM, and only a small change in SNAI1 expression (Supplementary Figure 1B).

Having established the EMT system, we performed the AcceSssIble assay for samples collected at the 2-, 8-, and 12-day time points. Since the Feinberg group claimed that DNA methylation does not change during TGF-beta induced EMT in the murine system [24], we focused our initial analysis on the methylation changes induced by TWIST in our human system. On performing a non-supervised hierarchical clustering of the most variable probes across all time points, we observed that distinct groups of are methylated or demethylated genes are apparent in the TWIST expressing cells (Supplementary Figure 1C), demonstrating that changes in DNA methylation can accompany certain molecular transitions that result in EMT.

**Figure 1:**
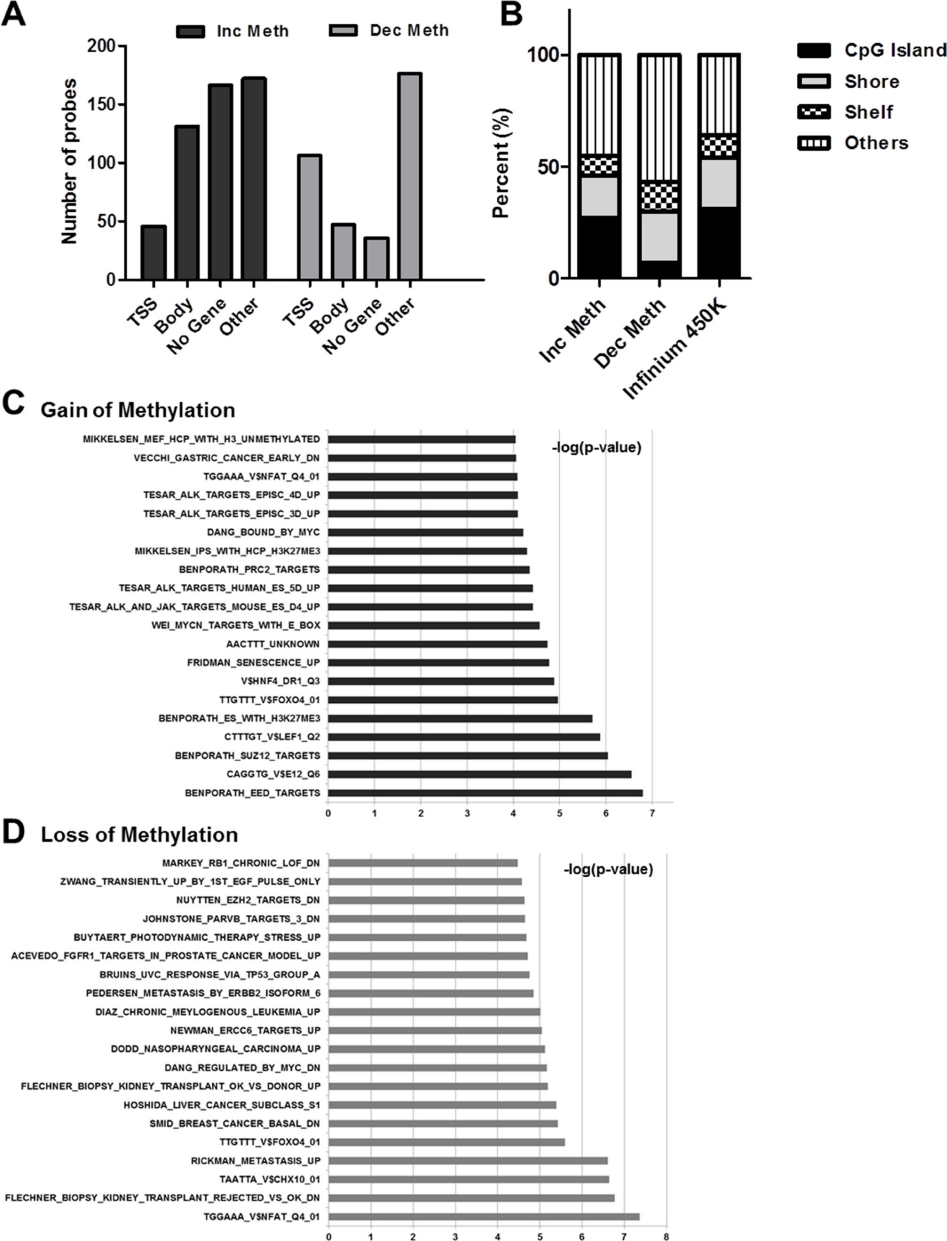
DNA methylation changes induced by TWIST-EMT are predominantly demethylation events at the TSS, localized to non-CpG islands (**A**) Quantification of probes that are demethylated and that attain methylation > 0.2 delta-beta in different genomic regions (**B**) Breakdown of TSS probes in relation to CpG island status (**C** and **D**) GSEA of gain and loss of methylated genes

We then took a closer look at DNA methylation changes that occur in the TWIST-induced system and quantified them as gain or loss of methylation events. Notably, while the total numbers of increase and decrease in methylation events were similar, the number of decrease in methylation events at the TSS was 2-fold greater than the increase in methylation events (Figure 1A). Of note, there are higher gene body DNA methylation increase than gene body DNA methylation decrease probes (Figure 1A), supporting the TSS methylation patterns – since gene body methylation shows the reverse effect as TSS methylation [28].

The TSS group of genes that displayed methylation changes were further classified as probes in CpG islands, in island related areas such as the shore and shelf regions and in non-island TSS regions. Interestingly, we found that more than 50% of the methylation changes occurred in non-island regions and only a minority of changes occurred in the CpG island itself (Figure 1B), despite the over-representation of CpG island probes on the methylation array. This indicates that methylation at non-island genes is more impacted during EMT progression. This finding corroborates the methylation changes observed in the developmental context, wherein DNA methylation changes at non-island genes such as OCT4 and NANOG are the key events in differentiation and in reprogramming [29]. We then performed GSEA (gene set enrichment analysis), using the Molecular Signatures Database (MSigDB) of gene groups that showed increased and decreased methylation (Figure 1C and 1D). Notably, we found that genes that gained methylation were enriched for E-box motifs at the promoters (*p*-value = 2.7e^-5^), suggesting a potential targeting phenomenon by TWIST, which is known to bind to this motif [30]. In addition, the group that lost methylation was enriched for pro-metastatic genes (*p*-value = 2.5e^-7^), supporting the role of EMT in metastasis. Further, in order to assess whether the changes in DNA methylation events were associated with chromatin changes, we analyzed the group of genes that gained methylation on day 2 in the TWIST-induced system and found that methylation patterns were retained over time (Supplementary Figure 1D). Several of these genes (top of each column in the heatmap) attained a functional gain of DNA methylation, wherein methylation was accompanied by closing of chromatin from an open configuration. Together, our results demonstrate that TWIST-induced EMT is accompanied by changes in DNA methylation, majority of which lie in non-CpG island promoters, in contrast to the observations made in the TGF-beta induced mouse system. The DNA demethylation events have a functional significance in EMT, enriching for genes with known metastasis-related behavior, and displaying a change in chromatin status.

### Chromatin accessibility alterations occur independent of and dependent on DNA methylation changes during EMT

**Figure 2:**
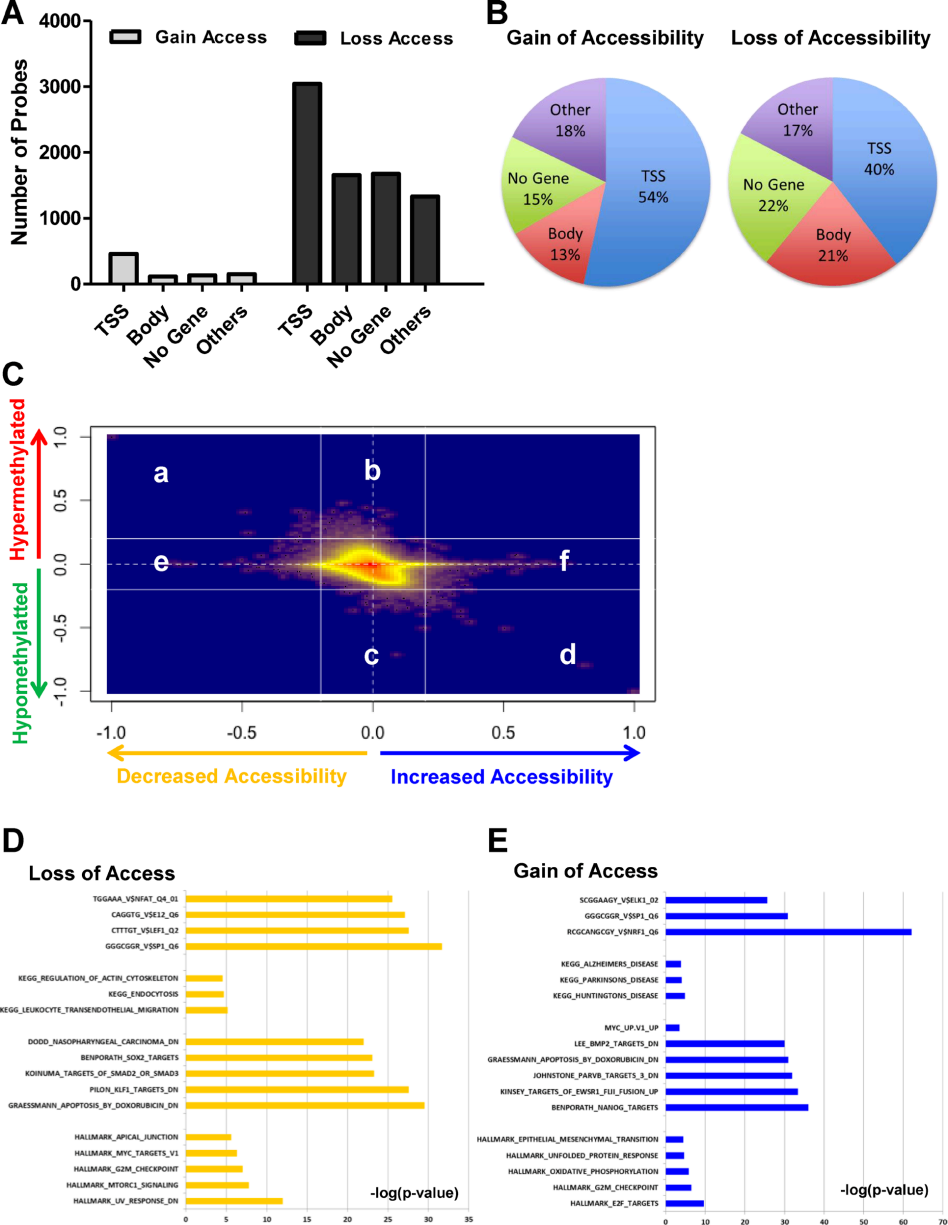
Large proportions of accessibility changes occur at TSS sites and accessibility changes are seen dependent and independent of DNA methylation during EMT (**A**) Quantification of probes that get increased accessibility and decreased accessibility at any time point > 0.3 delta-beta in different genomic regions. (**B**) Pie charts depicting the occurrence of gain and loss of accessibility changes with respect to genomic context for TWIST-induced EMT shows that a large proportion of changes localize to the TSS. (**C**) Smooth scatter plots of delta-methylation (methylation in treated – methylation in control) and delta-accessibility (accessibility in treated – accessibility in control) for day 2 in TWIST-overexpressing cells. (**D** and **E**) GSEA of gain and loss of accessibility genes

Next, we took a closer look at the location of the probes that display gain and loss of accessibility events within the genomic context – focusing on TSS sites, body regions, gene deserts and other gene related regions (such as 5′UTR, first exon etc.). While the number of loss of accessibility events was higher than gain of accessibility events (Figure 2A), we found that changes in the TSS sites were the largest in proportion to all changes in TWIST-induced EMT, particularly gain of accessibility changes (Figure 2B), suggesting that this accessibility change could directly affect the transcription state of genes.

To study the spectrum of epigenetic changes using the metrics of DNA methylation and chromatin accessibility changes, we plotted changes in methylation, methylation in the treated minus the control (Delta Methylation), against the change in accessibility in the treated minus the control (Delta Accessibility) as a kernel density scatter plot for all three time points during TWIST-driven EMT in HMECs (Figure 2C, Supplementary Figure 2A and 2C). Region a and d represent DNA methylation dependent changes, while region e and f comprise of gene regions that are DNA methylation-independent. Region b and c represent the DNA methylation changes without chromatin accessibility changes and may represent a passenger effect based on our previous report [20]. We therefore focused on regions a, d, e and f. Notably, TWIST-induced EMT results in changes in all groups with accessibility changes, dependent on and independent of DNA methylation at all-time points (Figure 2C, Supplementary Figure 2A and 2C). We catalogued the genes that harbored chromatin accessibility changes into 6 groups depending on the time point and the nature of chromatin change (i.e., closure or opening) (Supplementary Table 1) and posited that these are “functional changes”, since chromatin state was altered.

To assess the functional importance of increased and decreased chromatin accessibility groups during TWIST driven EMT, we focused on early time points (day 2) and performed GSEA (Figure 2D and 2E). Since the link between promoter and gene expression is straight forward, we used TSS probes in this study. Of note, gene sets with both types of accessibility changes showed enrichment of known EMT related pathways such as upstream SP1 binding motif, MYC targets etc. [2, 31, 32], suggesting that the change in chromatin accessibility is a potential functional driver of EMT. As expected, genes with decreased accessibility were enriched for actin-cytoskeletal regulation and trans-endothelial migration (Figure 2D), further validating our results from this methodology. Interestingly, genes that demonstrated increased accessibility were enriched for neurodegenerative disorders such as Alzheimer’s, Parkinson’s, and Huntington's disease-related pathways (Figure 2E). This opens up avenues for future research towards understand how these genes affect disease progression and their potential relationship with EMT. Further, we performed functional annotation analysis of intermediate (day 8) and late (day 12) chromatin accessibility genes, showing that loss of access gene groups were enriched for cell adhesion and gain of access gene groups were enriched for cell cycle regulation, respectively (Supplementary Figure 2B and 2D). Together, our results demonstrate that chromatin accessibility changes occur in addition to DNA methylation changes during EMT; regions with chromatin changes correspond to genes that are potentially critical drivers of the process.

### TRIM29 identified as a novel marker of EMT

To identify putative novel driver genes, we narrowed down the list of genes that gain or lose accessibility at the TSS during early time points by overlapping with the “core EMT signature” list obtained previously by using 5 different methods of EMT induction in the HMEC system [33]. Unexpectedly, very few genes were found to intersect; 14 intersecting genes were found in the decreased accessibility/downregulated group and 4 genes were found in the increased accessibility/upregulated group (Table 1). We further confirmed with two downregulated genes (EPHA1, IRF6) and one upregulated gene (FADS1) by real time PCR (Supplementary Figure 3A). VIM, a known player in EMT, which gains expression in the combined lists, also displays chromatin opening at day 2 (Table 1 and Supplementary Figure 3B). The central determinant of epithelial fate, CDH1, displays chromatin closing at day 8 (Supplementary Table 1) along with decreased expression (Supplementary Figure 1B), validating that our method of querying chromatin accessibility changes is capable of identifying driver genes.

**Table 1:** Chromatin accessibility study identifies driver candidates during EMT

Accessibility change Day 2	Gene expression change	Gene	Gene full name	GO Function
**Decreased (closed)**	**Down-regulated**	KRT5	keratin 5	scaffold protein binding and structural constituent of cytoskeleton
		IRF6	interferon regulatory factor 6	regulatory region DNA binding and sequence-specific DNA binding transcription factor activity
		MAP7	microtubule-associated protein 7	structural molecule activity and receptor binding.
		PTPN3	protein tyrosine phosphatase, non-receptor type 3	protein tyrosine phosphatase activityand ATPase binding
		SNCA	synuclein, alpha	phospholipid binding and calcium ion binding.
		MST1R	macrophage stimulating 1 receptor	macrophage colony-stimulating factor receptor activity andenzyme binding
		CYP27B1	cytochrome P450, family 27	electron carrier activity and heme binding
		EPHA1	EPH receptor A1	Trans membrane-ephrin receptor activity and protein kinase binding
		CYP4F11	cytochrome P450, subfamily 4F, polypeptide 11	electron carrier activity and heme binding
		GNAL	guanine nucleotide binding protein	GTP binding and GTPase activity
		DDR1	discoidin domain receptor tyrosine kinase 1	collagen binding and protein tyrosine kinase collagen receptor activity
		IL1RN	interleukin 1 receptor antagonist	interleukin-1 receptor binding and interleukin-1, Type I receptor binding
		WWC1	WW and C2 domain containing 1	transcription coactivator activity and protein binding, bridging
		**TRIM29**	**tripartite motif containing 29**	**p53 binding and sequence-specific DNA binding transcription factor activity**
**Increased (opened)**	**Up-regulated**	FADS1	fatty acid desaturase 1	oxidoreductase activity and iron ion binding
		RGS4	regulator of G-protein signaling 4	calmodulin binding and GTPase activator activity
		ACP1	acid phosphatase 1	SH3 domain binding and non-membrane spanning protein tyrosine phosphatase activity
		VIM	vimentin	structural constituent of cytoskeleton and identical protein binding

Intersection of genes that gain or lose accessibility on day 2 of TWIST-driven EMT with lists of genes getting upregulated or downregulated upon EMT induction with 5 different methods [33].

**Figure 3:**
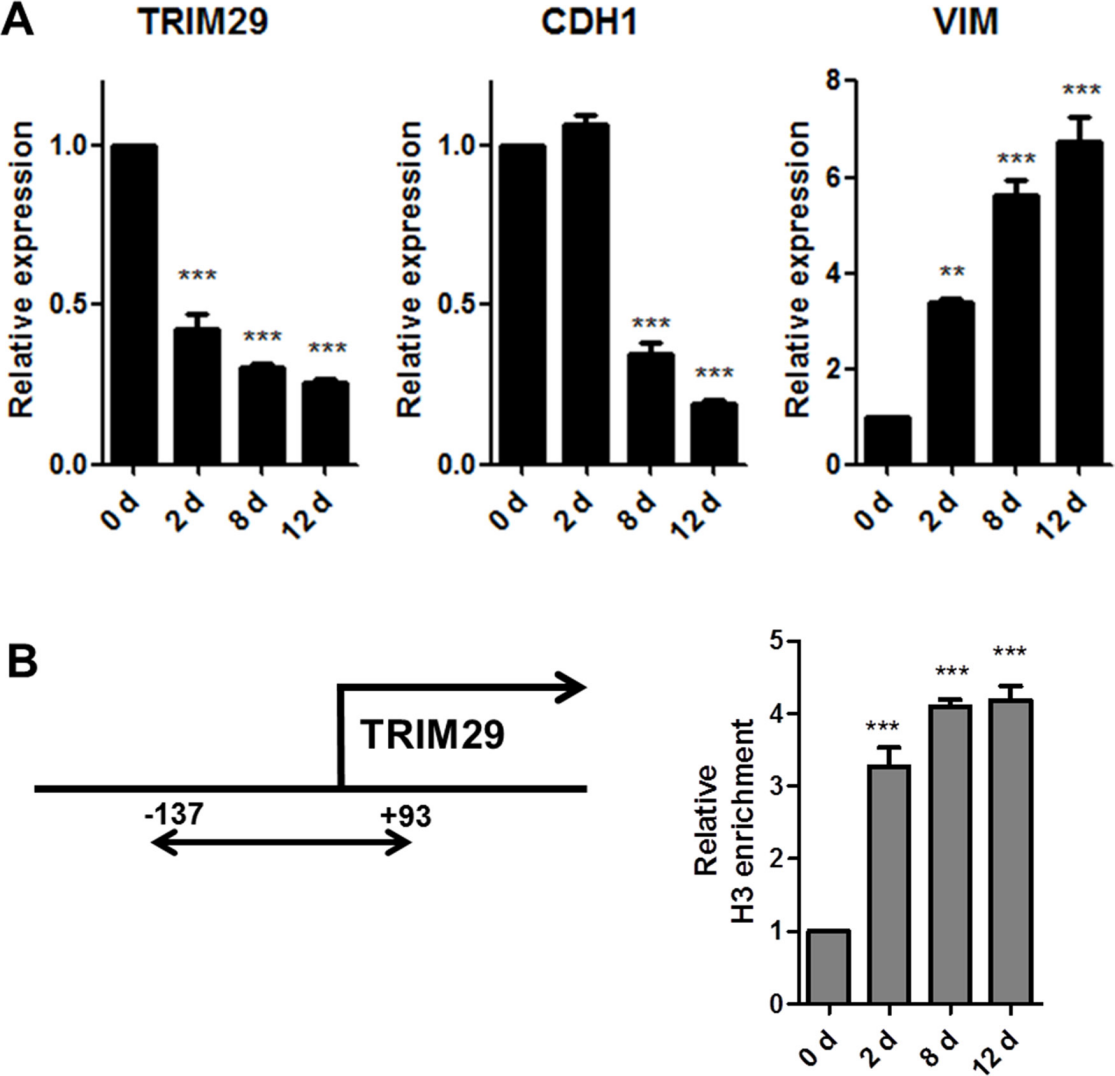
Identification of TRIM29 by the AcceSssIble assay and validation of predictions (**A**) Real time RT-PCR analysis for TRIM29, CDH1 and VIM expression was performed during TWIST driven EMT. Downregulation of TRIM29 and CDH1 and upregulation of VIM were observed which correlated with prediction of accessibility changes. (**B**) ChIP assay for histone H3 was performed at the promoter of TRIM29 and its decreased accessibility was confirmed with increased histone H3 enrichment. Data are presented as the mean ± S.E.M. (^**^p < 0.01, ^***^p < 0.001 vs. Control).

Next, we focused on the group of genes that showed decreased accessibility during the transition, since there is a dearth of information on epithelial genes whose loss drives EMT and the genes that are most studied, gain expression and block the epithelial fate. TRIM29 caught our attention on the list of genes that close during EMT. TRIM29 encodes a protein that belongs to the zinc finger family and contains multiple zinc finger motifs and a leucine zipper motif [34]. It has been implicated as a tumor suppressor in breast cancers [35] and as an oncogene in several cancers through negative regulation of p53 via unknown mechanisms [34, 36, 37]. Furthermore, Ai and colleagues very recently reported that TRIM29 suppresses TWIST and EMT [38]. Since we used a TWIST driven EMT model system, we proceeded to investigate the interconnection between TRIM29 and TWIST. As a first step, we confirmed TRIM29 mRNA expression by real-time PCR along with CDH1 and VIM (Figure 3A). Corroborating our chromatin accessibility results, downregulation of TRIM29 and upregulation of VIM is observed on day 2, while decreased CDH1 is detected on day 8 (Figure 3A and Supplementary Figure 3B). Chromatin configuration of TRIM29 promoter was also confirmed by chromatin immunoprecipitation (ChIP) assay using a histone H3 antibody (Figure 3B).

### Loss of TRIM29 mediates EMT

**Figure 4:**
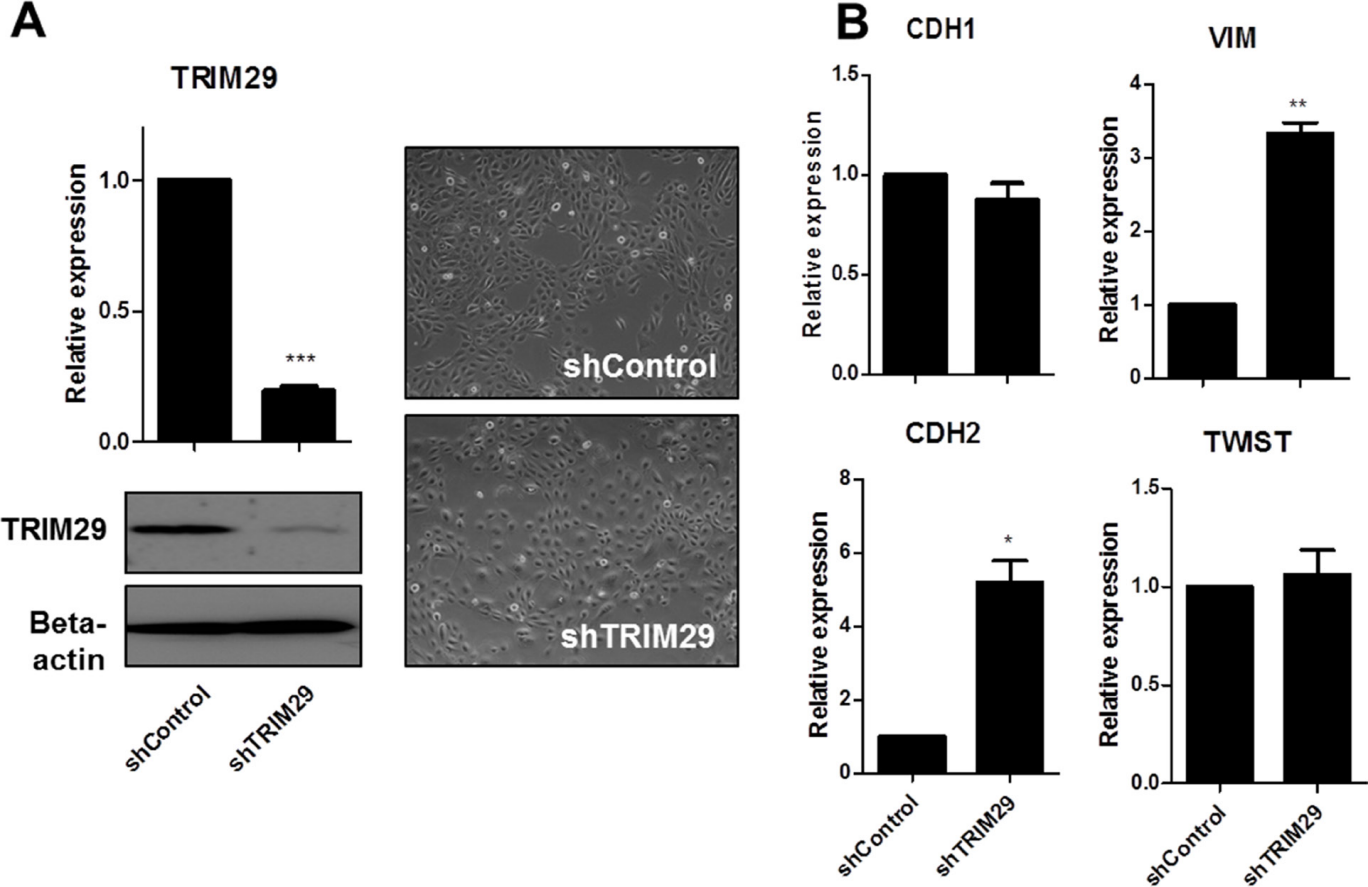
Loss of TRIM29 induces upregulation of CDH2 and VIM but little change in CDH1 and TWIST expression (**A**) Stably infected TRIM29 knocked down HMECs were selected with puromycin. Real time RT-PCR and western blot confirmed the knockdown of TRIM29. Phase-contrast images of TRIM29 knocked down HMECs are shown with control cells. (^***^p < 0.001 vs. Control). (**B**) CDH1, CDH2, VIM and TWIST mRNA levels were determined by real-time PCR after stable knockdown of TRIM29. Data are presented as the mean ± S.E.M. (^*^p <0.05, ^**^p < 0.01 vs. Control).

To test whether the loss of TRIM29 was sufficient to drive an EMT phenotype, we generated stable TRIM29 knockdown cells using shRNA transduction and confirmed its suppression by real-time PCR and western blot. We also observed that the transduced cells displayed a change in morphology and showed a mild mesenchymal-like phenotype with loss of cell-cell contact (Figure 4A). We detected increased VIM and CDH2 mRNA expression after knockdown of TRIM29 but little change in CDH1 and TWIST (Figure 4B).

**Figure 5:**
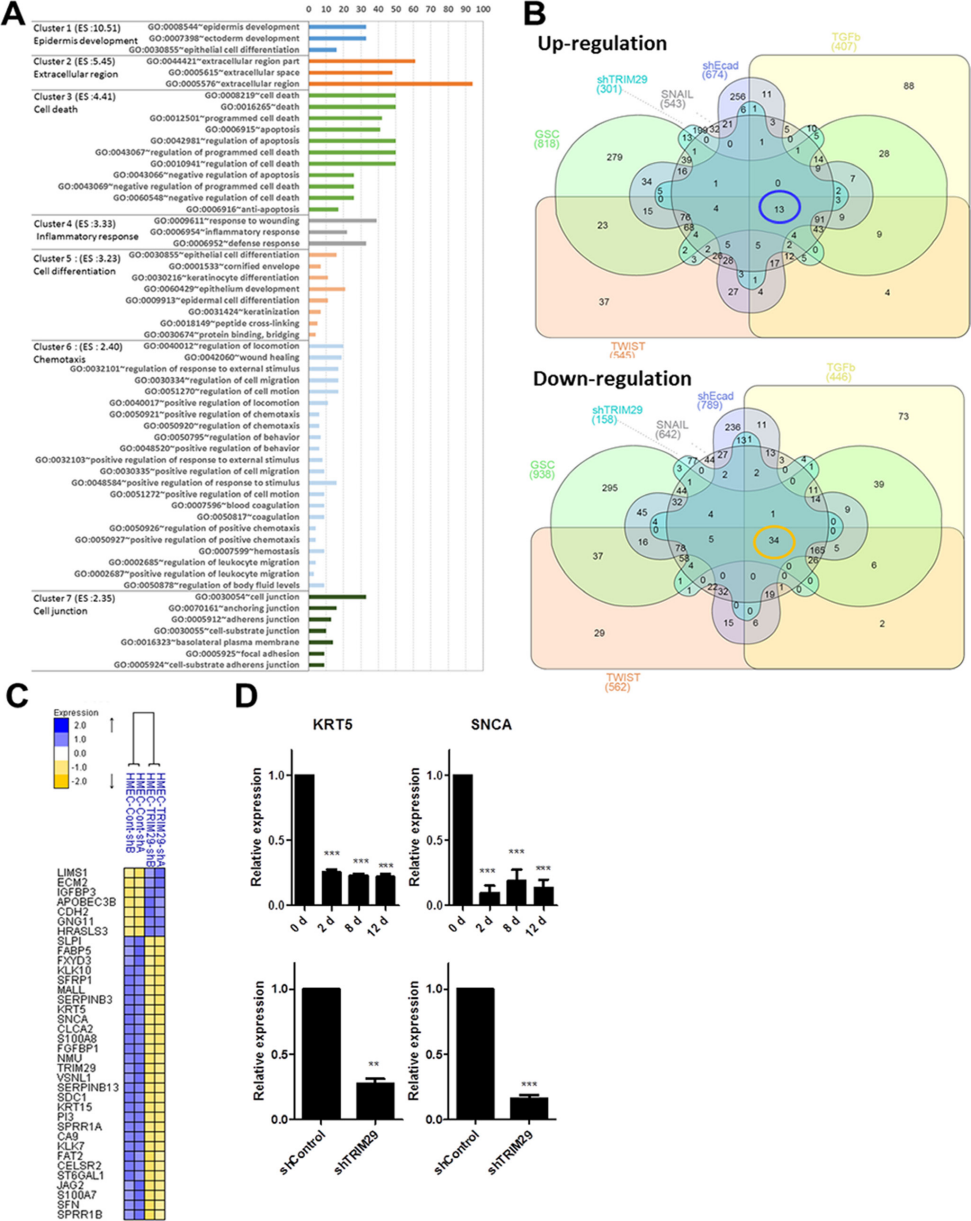
Loss of TRIM29 mediates EMT (**A**) The graphs show GO terms significantly associated with TRIM29 knockdown in HMEC. Bars correspond to the percentage of each GO term. (**B**) Venn diagrams of genes showing up-(upper) and down-(lower) regulation in the core EMT signature with TRIM29 knockdown. (**C**) Heatmap of refined EMT mediators (**D**) Real time RT-PCR analysis for KRT5 and SNCA expression was performed during TWIST driven EMT and after stable knockdown of TRIM29.

To comprehensively understand the role of TRIM29 in mediating EMT in HMECs, we performed and analyzed gene expression microarrays following knockdown of TRIM29 (Figure 5 and Supplementary Figure 4A). Loss of TRIM29 induced expression changes in a relatively small subset of genes. Therefore, we used a cutoff of 1.5 fold expression change at a 0.05 significance level to identify the affected genes (Supplementary Figure 4A). To assess the role of TRIM29 in HMECs, we performed Gene-Enrichment and Functional Annotation analysis for the identified genes and found multiple GO categories associated with TRIM29 (Figure 5A). Of interest, the genes in cluster1 are highly enriched for epidermal development. Other identified genes affect extracellular regions, cell death, inflammatory response, cellular differentiation, chemotaxis, and cell junctions (Figure 5A), suggesting that TRIM29 could be a key mediator of EMT and other important cellular activities.

In order to understand the significance of TRIM29 loss, we reanalyzed the “core EMT signature” (220 genes as shown in Supplementary Figure 4B) with genes identified to be downstream of TRIM29. After screening out the common genes since both experiments used different microarray chips, we separated the genes into two groups – upregulated or downregulated expression (Figure 5B). We identified 34 downregulated genes and 13 upregulated genes in common (Figure 5B and Supplementary Table 2). We then applied the *p* value < 0.05 and finally proposed 36 genes as refined EMT mediators (Figure 5C). Notably, two genes (KRT5 and SNCA) that belonged to our list of EMT driver candidates in Table 1 emerged in this analysis as well. KRT5 (keratin 5) is a scaffold protein and hence, presumably important for epithelial fate [39]. SNCA encodes synuclein-alpha which is closely related to several neurodegenerative diseases such as dementia and Parkinson's disease [40]. However, to our knowledge, its role in EMT is not reported. We confirmed the expression levels of these two genes expression level during TWIST driven EMT and after knockdown of TRIM29 (Figure 5D). It would be interesting to further study the role of these genes during EMT and the role of EMT in neurodegenerative diseases.

## DISCUSSION

There is no doubt that EMT necessitates chromatin remodeling in order to attain the appropriate configuration to enhance or repress expression of the plethora of genes that drive the process. Genetic aspects of the EMT process have been extensively studied, but the factors that mediate these changes are yet to be determined. Further, the epigenetic aspect of EMT is a crucial area that needs exploration. The only comprehensive epigenomic study to date conducted by the Feinberg group demonstrated that no DNA methylation changes occurred during TGF-beta driven EMT in the mouse system [24]. In order to extend this study to the human system, we performed AcceSssIble to coordinately study DNA methylation and chromatin accessibility changes during TWIST-induced EMT in a human mammary epithelial cell line. Using our methodology, we were able to screen for and validate functional epigenetic changes that are novel markers of the TWIST driven EMT process. We also found that the TWIST system resulted in DNA demethylation and gain of methylation at several loci. Regions showing loss of methylation were enriched for metastasis-related genes, providing biological justification for demethylation at these loci (Figure 1). This result demonstrated that the different pathways to attain EMT are each accompanied with very different epigenetic changes. We identified both chromatin opening and closing events, predominantly independent of DNA methylation (Figure 2). TWIST-induced EMT displayed more chromatin closing changes, in contrast with the previous report, which implicated open chromatin events as being crucial to the mesenchymal transformation of epithelial cells [24]. Although both processes result in the conversion of cells to a mesenchymal like appearance, the epigenetic changes that mediate these processes appear to be different. The differences in epigenetic changes that occur during the two systems of EMT raise two intriguing possibilities, that there are many paths to the epigenomic remodeling essential for this cell-type conversion or that there exist fundamental species specific mechanisms.

Hypothesizing that the driver epigenetic changes in EMT were those that involved a chromatin accessibility change at early time points, we analyzed the TWIST-induced EMT, and screened for putative driver genes by further overlapping with the “core EMT signature” and identified TRIM29 (Table 1 and Figure 3). It has been reported that knockdown of TRIM29 in breast cancer cells was sufficient to induce EMT with increased level of CDH2 and VIM and decreased level of CDH1 via suppression of TWIST [38]. When we knocked down TRIM29 in non-transformed HMEC, we found that its suppression was sufficient to allow the upregulation of CDH2 and VIM, but had little effect on CDH1 and TWIST levels (Figure 4). This discrepancy might reflect a context dependent molecular function of TRIM29. Further investigation is required to understand the underlying mechanism.

Ontological analysis revealed that the potential targets of TRIM29 are highly enriched in EMT related cellular pathways such as epidermal development, cellular differentiation and cell junctions (Figure 5A), suggesting that TRIM29 is a key mediator of EMT. Next, we scrutinized the “core EMT signature” against the identified TRIM29 targets and narrowed down the refined EMT mediators (Figure 5B and Supplementary Table 2). Our analysis resulted in the identification of 2 potential novel drivers of EMT, KRT5, and SNCA, in addition to 36 other genes as potentially robust markers of EMT. It would be interesting to investigate the role of TRIM29 that has been implicated in blocking pathogenesis, be it by maintaining the epithelial fate, as a tumor suppressor, or in preventing neurodegenerative disorders.

To date, the mechanism by which TRIM29 affects downstream genes remains an open question but its role in cellular process such as tumorigenesis is known to be contextual [34, 36–38, 41]. It is known that TRIM29 negatively regulates P53 and functions as an oncogene [36, 42] and P53 inhibits the reverse process of EMT [43]. To investigate the relationship with P53, we performed GSEA with the hallmark of the P53 pathway and found a very mild negative correlation (NES -1.13) which was not significant (Supplementary Figure 4C). However, further study is required to determine a definitive link between TRIM29 and P53. While exogenous TRIM29 has been shown to be localized in cytoplasm, very recently endogenous TRIM29 was found to be a histone binding protein and was shown to act as a scaffold protein responsible for the DNA damage response [44]. Inaccuracy of DNA damage repair leads to various diseases including cancer, neurodegeneration and immunodeficiency [45]. Of note, genes that demonstrated increase in chromatin accessibility early during EMT were highly enriched in Alzheimer's and Parkinson's disease (Figure 2E). Further, SNCA, a gene notorious for involvement in such diseases surfaced as a high probability EMT marker from our analysis of downstream targets of TRIM29 (Figure 5). It will be intriguing and important to understand the role of these genes during EMT and how these genes or the EMT process affects the progression of these diseases.

Much remains to be understood about the epigenomics of EMT especially in human systems. In this study, we were able to coordinately study DNA methylation and chromatin accessibility changes in a TWIST driven EMT system and used this to identify TRIM29 as a key mediator of the process along with 18 driver candidates. The results, obtained using loss of TRIM29 followed by gene expression arrays and further scrutinizing the previously reported “core EMT signature,” allowed us to identify high-probability EMT markers. Our results have powerful implications for future basic and clinical research in this developmental process and its resurfacing during progression of diseases such as cancer and neurodegenerative diseases.

## MATERIALS AND METHODS

### Cell culture

The immortalized human mammary epithelial cell line (HMEC) and the derivative cell line HMEC-TWIST-ER were kindly provided by the Robert Weinberg laboratory (MIT). Cells were cultured as described previously [46]. To induce TWIST expression, HMEC-TWIST-ER cells were continuously exposed to 4-hydroxy tamoxifen (4-OHT) at a concentration of 20 nM until morphological change was observed, typically at 12 days.

### M.SssI treatment and DNA methylation array

For extraction of nuclei, cells were trypsinized and centrifuged for 5 min at 200 × *g*, then washed with ice-cold PBS and resuspended in 1 ml of ice-cold nuclei buffer (10 mM Tris, pH 7.4, 10 mM NaCl, 3 mM MgCl_2_, 0.1 mM EDTA and 0.5% NP-40, and protease inhibitors) per 5 × 10^6^ cells and incubated on ice for 5 min. Nuclei were recovered by centrifugation at 900 × *g* for 3 min and washed in nuclei wash buffer (10 mM Tris, pH 7.4, 10 mM NaCl, 3 mM MgCl_2_ and 0.1 mM EDTA containing protease inhibitors). Fresh nuclear preparations (2 × 10^5^ cells) were resuspended in 1×M.SssI reaction buffer (NEB), then treated with 50U of M.SssI (NEB) with 15 μl 10× reaction buffer, 45 μl 1M sucrose, and 0.75 μl S-Adenosyl methionine (SAM) in 150 μl volume. Reactions were quenched by an equal volume of stop solution (20 nM Tris-HCl [pH 7.9], 600 mM NaCl, 1% SDS, 10 mM EDTA and 400 μg/ml proteinase K) and incubated overnight at 55°C (23). DNA was purified by phenol/chloroform extraction and ethanol precipitation. Bisulfite conversion was performed using the EZ DNA methylation kit (Zymo Research). All samples passed bisulfite conversion quality control, and were subsequently processed for the Illumina Infinium DNA methylation platform (Human Methylation 450 Bead Chip). For validation of enzyme treatment and local accessibility, molecules were cloned using the Topo TA Kit (Invitrogen) and sequenced.

### Global DNA methylation and chromatin accessibility analysis

Global DNA methylation and chromatin accessibility analysis was carried out as described previously [20]. The Infinium HumanMethylation450 array (Illumina, Inc.) was used to analyze bisulfite-treated DNA. Gene promoters with probes having a beta value > 0.7 were considered as methylated and < 0.3 were considered as unmethylated, and probes with intermediate values were partially methylated. The accessibility scale was defined as the difference between the beta value of M.SssI-treated cells minus the beta value of the no-enzyme control (delta-beta), defined on a scale of 0–1, after removing the probes with delta-beta < 0. Accessible probes were defined as those having delta-beta > 0.2 using standard receiver-operating characteristic curve analysis. Heat maps were generated using R package gplots and the density plots were generated using R package ggplot2.

### RNA extraction and reverse transcription PCR

Total RNA was extracted using TRIzol reagent, digested with DNase I, and reverse transcribed using a High Capacity cDNA Reverse Transcription Kit (Applied Biosystems). Amplification of cDNA was performed on a LightCycler^®^ 480 II (Roche) using the LightCycler^®^ 480 SYBR Green I Master mix (Roche), according to the recommended conditions. cDNA was amplified using the following gene-specific primers:

RT_TRIM29: 5′-GATGCTGTGGACCAAGTGAA, 3′-TGCTCATCAATGCACCAAAT; RT_CDH1: 5′-TAG AGGGTCACCGCGTCTAT, 3′-TCACAGGTGCTTTG CAGTTC; RT_VIM: 5′-TACGGACGTCGAGTTTCCTC, 3′-GAAACTTCTGCAGCCTTTGG; RT_TWIST: 5′-GTC CGCAGTCTTACGAGGAG, 3′-TGGAGGACCTGGTA GAGGAA; RT_ EPHA1: 5′-GTGGACACTGTCATAGG AGAAGG, 3′-GGTCTTAATGGCCACAGTCTTG; RT_KRT5: 5′-CGCAACCTGGACCTGGATAG, 3′-GGCT CTCAGCCTCTGGATCA; RT_SCNA: 5′- AGGACTT TCAAAGGCCAAGG, 3′- TCCTCCAACATTTGTCA CTTGC; RT_GAPDH: 5′-GAGTCAACGGATTTGG TCGT, 3′-TGGAAGATGGTGATGGGATT.

### Western blot analysis

Cells were lysed with RIPA buffer (150 mM NaCl, 1.0% NP-40, 0.5% sodium deoxycholate, 0.1% SDS, 50 mM Tris-HCl (pH 8.0), and protease inhibitors) and sonicated briefly (30% amplitude, 3 sec). Cell lysates were boiled in Laemmli sample buffer for 3 min, protein concentration was measured by Bradford protein assay, and 30 μg of each protein sample was subjected to SDS-PAGE. Proteins were transferred to polyvinylidene difluoride membranes; the membranes were blocked for 30 min in Tris-buffered saline (TBS) containing 0.1% Tween 20 and 5% (w/v) dry skim milk powder, and incubated overnight with primary antibodies against TRIM29 (Cell Signaling, cat#5182S) or beta-actin (Cell Signaling, cat#49675) (dilution ratio 1:1,000). The membranes then were washed with TBS-0.1% Tween 20, incubated for 1 h with a secondary antibody (dilution ratio 1:10,000), and visualized using an enhanced chemiluminescence detection kit (Amersham Life Sciences) after exposure on an LAS-3000 image detection system (Fuji).

### Chromatin immunoprecipitation (ChIP) assay

Chromatin immunoprecipitation (ChIP) assays were performed according to the instructions provided by Upstate Biotechnology. For each ChIP assay, 10 μg of DNA sheared by a sonicator, was pre-cleared with salmon-sperm DNA-saturated protein A Sepharose and then precipitated with H3 antibody (Abcam). After immunoprecipitation, the recovered chromatin fragments were subjected to real-time polymerase chain reaction. IgG control experiments were performed for all ChIPs and were accounted for in the IP/Input by presenting the results as (IP−IgG)/(Input−IgG). Primer sequences: TRIM29 promoter: 5′-GCGTGGTTCCTGTGCAATTA, 3′-TGCTGGGGTTCAGGATAGGT

### shRNA transduction

shTRIM29 constructs were purchased from Sigma-Aldrich. MISSION lentiviral packaging mix was used for lentivirus production. Infected derivative cells stably expressing shRNA were selected with 1.25 μg/mL puromycin.

### Gene expression microarray

Total RNA was amplified and purified using TargetAmp-Nano Labeling Kit for Illumina Expression BeadChip (EPICENTRE, Madison, USA) to yield biotinylated cRNA according to the manufacturer's instructions. Briefly, 80 ng of total RNA was reverse-transcribed to cDNA using a T7 oligo (dT) primer. Second-strand cDNA was synthesized, transcribed *in vitro*, and labeled with biotin-NTP. After purification, the cRNA was quantified using the ND-1000 Spectrophotometer (NanoDrop, Wilmington, USA). Hybridization and data export; 750 ng of labeled cRNA samples were hybridized to each Human HT-12 v4.0 Expression Beadchip for 17 h at 58°C, according to the manufacturer's instructions (Illumina, Inc., San Diego, USA). Detection of array signal was carried out using Amersham fluorolink streptavidin-Cy3 (GE Healthcare Bio-Sciences, Little Chalfont, UK) according to the bead array manual. Arrays were scanned with an Illumina bead array Reader confocal scanner according to the manufacturer's instructions. The quality of hybridization and overall chip performance were monitored manually by visual inspection of both internal quality control checks and the raw scanned data. Raw data were extracted using the software provided by the manufacturer (Illumina GenomeStudio v2011.1 (Gene Expression Module v1.9.0)). Array probes were logarithm-transformed and normalized by the quantile method.

### Statistical analysis

Results were expressed as mean ± SEM. Most statistical comparisons were calculated by one-way ANOVA followed by Bonferroni's post hoc test using GraphPad Prism. A *p* value of < 0.05 was considered significant.

### Data access

GEO AcceSssIble data set; GSE63366. Gene expression microarray of TRIM29 knockdown; GSE71375.

## SUPPLEMENTARY MATERIALS FIGURES AND TABLES





## References

[1] Nieto MA (2013). Epithelial plasticity: a common theme in embryonic and cancer cells. Science.

[2] Thiery JP, Acloque H, Huang RY, Nieto MA (2009). Epithelial-mesenchymal transitions in development and disease. Cell.

[3] Tiwari N, Gheldof A, Tatari M, Christofori G (2012). EMT as the ultimate survival mechanism of cancer cells. Semin Cancer Biol.

[4] Thiery JP, Sleeman JP (2006). Complex networks orchestrate epithelial-mesenchymal transitions. Nat Rev Mol Cell Biol.

[5] De Craene B, Berx G (2013). Regulatory networks defining EMT during cancer initiation and progression. Nat Rev Cancer.

[6] Savagner P, Yamada KM, Thiery JP (1997). The zinc-finger protein slug causes desmosome dissociation, an initial and necessary step for growth factor-induced epithelial-mesenchymal transition. J Cell Biol.

[7] Baylin SB, Jones PA (2011). A decade of exploring the cancer epigenome - biological and translational implications. Nat Rev Cancer.

[8] Sharma S, Kelly TK, Jones PA (2010). Epigenetics in cancer. Carcinogenesis.

[9] Bai L, Morozov AV (2010). Gene regulation by nucleosome positioning. Trends Genet.

[10] Segal E, Widom J (2009). What controls nucleosome positions?. Trends Genet.

[11] You JS, Kelly TK, De Carvalho DD, Taberlay PC, Liang G, Jones PA (2011). OCT4 establishes and maintains nucleosome-depleted regions that provide additional layers of epigenetic regulation of its target genes. Proc Natl Acad Sci USA.

[12] Kelly TK, Liu Y, Lay FD, Liang G, Berman BP, Jones PA (2012). Genome-wide mapping of nucleosome positioning and DNA methylation within individual DNA molecules. Genome Res.

[13] Sadeh R, Allis CD (2011). Genome-wide “re”-modeling of nucleosome positions. Cell.

[14] Barski A, Cuddapah S, Cui K, Roh TY, Schones DE, Wang Z, Wei G, Chepelev I, Zhao K (2007). High-resolution profiling of histone methylations in the human genome. Cell.

[15] Schones DE, Cui K, Cuddapah S, Roh TY, Barski A, Wang Z, Wei G, Zhao K (2008). Dynamic regulation of nucleosome positioning in the human genome. Cell.

[16] Giresi PG, Kim J, McDaniell RM, Iyer VR, Lieb JD. (2007). FAIRE (Formaldehyde-Assisted Isolation of Regulatory Elements) isolates active regulatory elements from human chromatin. Genome Res.

[17] Kilgore JA, Hoose SA, Gustafson TL, Porter W, Kladde MP (2007). Single-molecule and population probing of chromatin structure using DNA methyltransferases. Methods.

[18] Taberlay PC, Kelly TK, Liu CC, You JS, De Carvalho DD, Miranda TB, Zhou XJ, Liang G, Jones PA (2011). Polycomb-Repressed Genes Have Permissive Enhancers that Initiate Reprogramming. Cell.

[19] You JS, De Carvalho DD, Dai C, Liu M, Pandiyan K, Zhou XJ, Liang G, Jones PA (2013). SNF5 is an essential executor of epigenetic regulation during differentiation. PLoS Genet.

[20] Pandiyan K, You JS, Yang X, Dai C, Zhou XJ, Baylin SB, Jones PA, Liang G (2013). Functional DNA demethylation is accompanied by chromatin accessibility. Nucleic Acids Res.

[21] Sandoval J, Heyn H, Moran S, Serra-Musach J, Pujana MA, Bibikova M, Esteller M (2011). Validation of a DNA methylation microarray for 450,000 CpG sites in the human genome. Epigenetics.

[22] Tam WL, Weinberg RA (2013). The epigenetics of epithelial-mesenchymal plasticity in cancer. Nat Med.

[23] Wu CY, Tsai YP, Wu MZ, Teng SC, Wu KJ (2012). Epigenetic reprogramming and post-transcriptional regulation during the epithelial-mesenchymal transition. Trends Genet.

[24] McDonald OG, Wu H, Timp W, Doi A, Feinberg AP (2011). Genome-scale epigenetic reprogramming during epithelial-to-mesenchymal transition. Nat Struct Mol Biol.

[25] Ginis I, Luo Y, Miura T, Thies S, Brandenberger R, Gerecht-Nir S, Amit M, Hoke A, Carpenter MK, Itskovitz-Eldor J, Rao MS (2004). Differences between human and mouse embryonic stem cells. Dev Biol.

[26] Fougerousse F, Bullen P, Herasse M, Lindsay S, Richard I, Wilson D, Suel L, Durand M, Robson S, Abitbol M, Beckmann JS, Strachan T (2000). Human-mouse differences in the embryonic expression patterns of developmental control genes and disease genes. Hum Mol Genet.

[27] Mani SA, Guo W, Liao MJ, Eaton EN, Ayyanan A, Zhou AY, Brooks M, Reinhard F, Zhang CC, Shipitsin M, Campbell LL, Polyak K, Brisken C (2008). The epithelial-mesenchymal transition generates cells with properties of stem cells. Cell.

[28] Yang X, Han H, De Carvalho DD, Lay FD, Jones PA, Liang G (2014). Gene body methylation can alter gene expression and is a therapeutic target in cancer. Cancer Cell.

[29] Wu SC, Zhang Y (2010). Active DNA demethylation: many roads lead to Rome. Nat Rev Mol Cell Biol.

[30] Villavicencio EH, Yoon JW, Frank DJ, Fuchtbauer EM, Walterhouse DO, Iannaccone PM (2002). Cooperative E-box regulation of human GLI1 by TWIST and USF. Genesis.

[31] Kolesnikoff N, Attema JL, Roslan S, Bert AG, Schwarz QP, Gregory PA, Goodall GJ (2014). Specificity protein 1 (Sp1) maintains basal epithelial expression of the miR-200 family: implications for epithelial-mesenchymal transition. J Biol Chem.

[32] Nam EH, Lee Y, Zhao XF, Park YK, Lee JW, Kim S (2014). ZEB2-Sp1 cooperation induces invasion by upregulating cadherin-11 and integrin alpha5 expression. Carcinogenesis.

[33] Taube JH, Herschkowitz JI, Komurov K, Zhou AY, Gupta S, Yang J, Hartwell K, Onder TT, Gupta PB, Evans KW, Hollier BG, Ram PT, Lander ES (2010). Core epithelial-to-mesenchymal transition interactome gene-expression signature is associated with claudin-low and metaplastic breast cancer subtypes. Proc Natl Acad Sci USA.

[34] Hatakeyama S (2011). TRIM proteins and cancer. Nat Rev Cancer.

[35] Liu J, Welm B, Boucher KM, Ebbert MT, Bernard PS (2012). TRIM29 functions as a tumor suppressor in nontumorigenic breast cells and invasive ER+ breast cancer. Am J Pathol.

[36] Yuan Z, Villagra A, Peng L, Coppola D, Glozak M, Sotomayor EM, Chen J, Lane WS, Seto E (2010). The ATDC (TRIM29) protein binds p53 and antagonizes p53-mediated functions. Mol Cell Biol.

[37] Zhou ZY, Yang GY, Zhou J, Yu MH (2012). Significance of TRIM29 and beta-catenin expression in non-small-cell lung cancer. J Chin Med Assoc.

[38] Ai L, Kim WJ, Alpay M, Tang M, Pardo CE, Hatakeyama S, May WS, Kladde MP, Heldermon CD, Siegel EM, Brown KD (2014). TRIM29 suppresses TWIST1 and invasive breast cancer behavior. Cancer Res.

[39] Stephens K, Ehrlich P, Weaver M, Le R, Spencer A, Sybert VP (1997). Primers for exon-specific amplification of the KRT5 gene: identification of novel and recurrent mutations in epidermolysis bullosa simplex patients. J Invest Dermatol.

[40] Stefanis L (2012). alpha-Synuclein in Parkinson's disease. Cold Spring Harb Perspect Med.

[41] Masuda Y, Takahashi H, Hatakeyama S (2015). TRIM29 regulates the p63-mediated pathway in cervical cancer cells. Biochim Biophys Acta.

[42] Kosaka Y, Inoue H, Ohmachi T, Yokoe T, Matsumoto T, Mimori K, Tanaka F, Watanabe M, Mori M (2007). Tripartite motif-containing 29 (TRIM29) is a novel marker for lymph node metastasis in gastric cancer. Ann Surg Oncol.

[43] Brosh R, Assia-Alroy Y, Molchadsky A, Bornstein C, Dekel E, Madar S, Shetzer Y, Rivlin N, Goldfinger N, Sarig R, Rotter V (2013). p53 counteracts reprogramming by inhibiting mesenchymal-to-epithelial transition. Cell Death Differ.

[44] Masuda Y, Takahashi H, Sato S, Tomomori-Sato C, Saraf A, Washburn MP, Florens L, Conaway RC, Conaway JW, Hatakeyama S (2015). TRIM29 regulates the assembly of DNA repair proteins into damaged chromatin. Nat Commun.

[45] Jackson SP, Bartek J (2009). The DNA-damage response in human biology and disease. Nature.

[46] Elenbaas B, Spirio L, Koerner F, Fleming MD, Zimonjic DB, Donaher JL, Popescu NC, Hahn WC, Weinberg RA (2001). Human breast cancer cells generated by oncogenic transformation of primary mammary epithelial cells. Genes Dev.

