# Supplementary material for: Epigenetic landscape change analysis during human EMT sheds light on a key EMT mediator TRIM29
Source: Oncotarget. 2017 Oct 9;8(58):98322–35. doi: 10.18632/oncotarget.21681 (PMC5716732; doi:10.18632/oncotarget.21681)
Supplement: Supplementary file 1 [file oncotarget-08-98322-s001.pdf]

Epigenetic landscape change analysis during human EMT sheds light on a key EMT mediator TRIM29

SUPPLEMENTARY MATERIALS

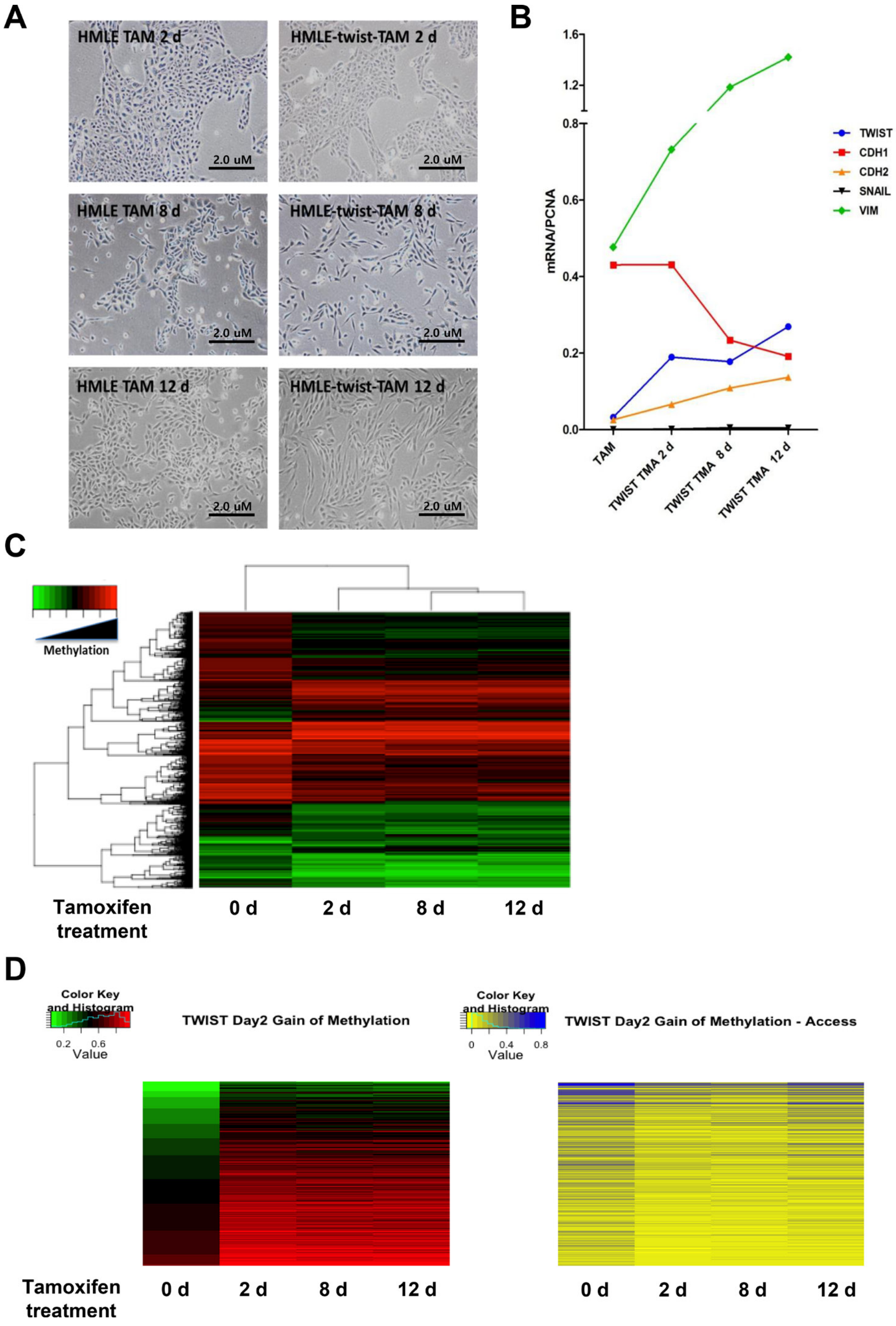

Supplementary Figure 1: (**A**) Phase-contrast images of HMEC expressing TWIST, and corresponding controls at days 2, 8 and 12 post-induction. EMT-like changes are apparent at day 8 in both systems. (**B**) Relative expression of the mRNAs encoding CDH1, CDH2, VIM, TWIST and SNAIL in HMEC induced to undergo EMT by TWIST induction, as determined by quantitative real-time PCR. GAPDH mRNA was used to normalize the variability in template loading. (**C**) Heatmap of the top 1 percent most variable probes on the platform, when compared across all samples for TWIST- driven EMT. Sustained gain and loss of methylation changes are observed during TWIST-EMT. (**D**) Heatmap and accessibility plot for all probes that gain methylation at day 2 post TWIST induction.

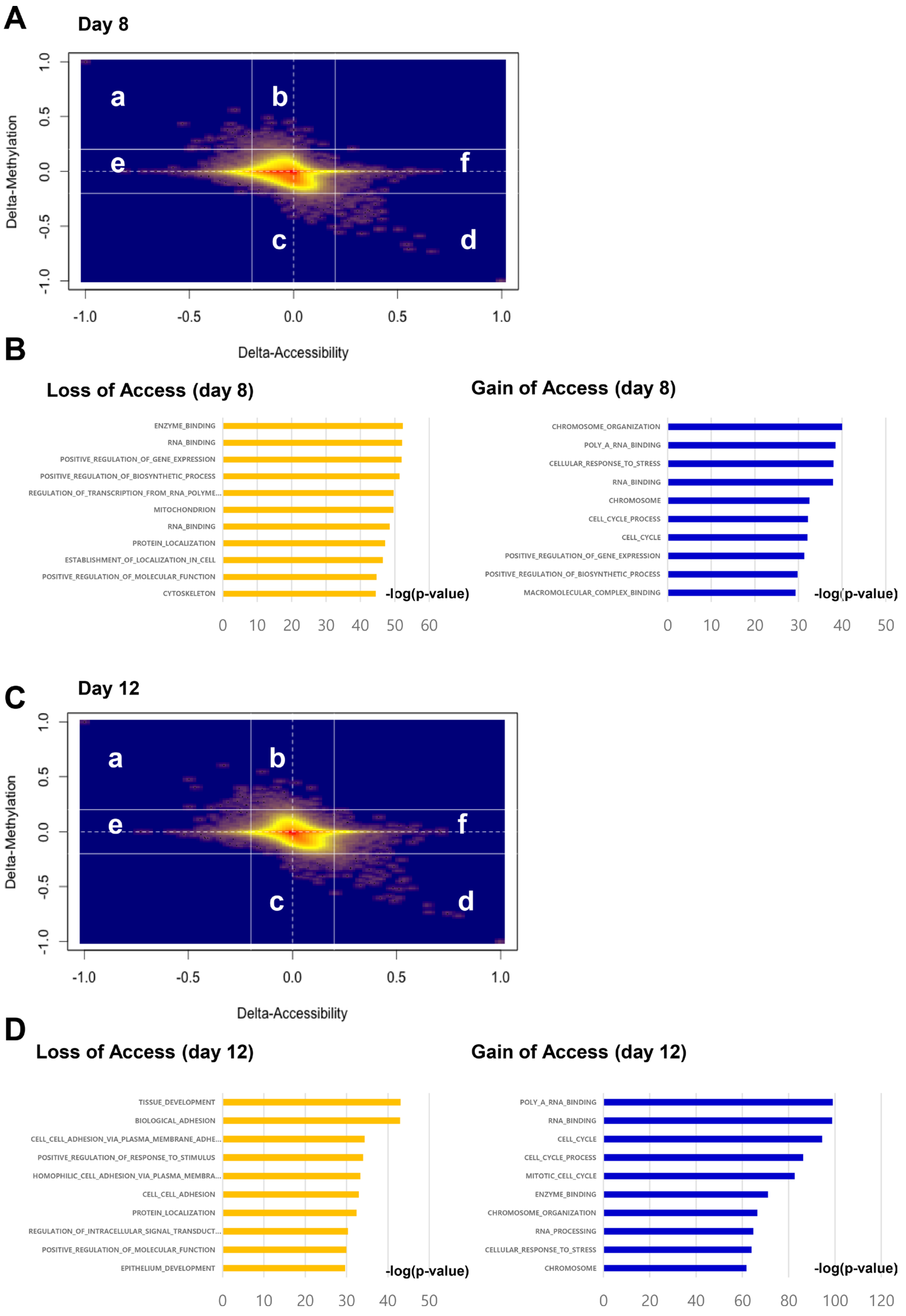

Supplementary Figure 2: (**A**) Smooth scatter plots of delta-methylation (methylation in treated – methylation in control) and delta-accessibility (accessibility in treated – accessibility in control) for day 8 for TWIST-overexpressing cells. (**B**) GSEA of gain and loss of accessibility genes for day 8. (**C**) Smooth scatter plots of delta-methylation (methylation in treated – methylation in control) and delta-accessibility (accessibility in treated – accessibility in control) for day 12 for TWIST-overexpressing cells. (**D**) GSEA of gain and loss of accessibility genes for day 12.

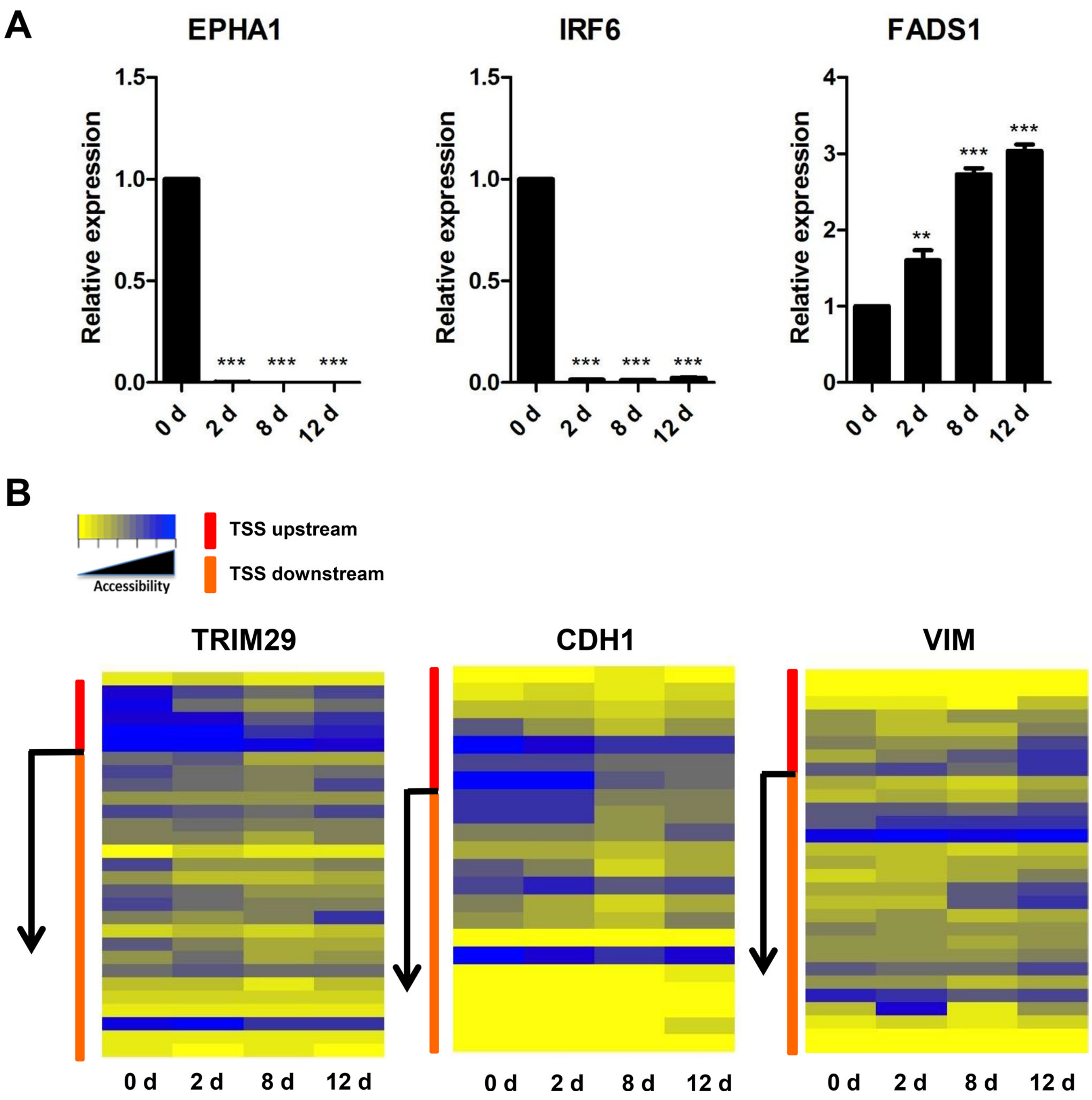

Supplementary Figure 3: (**A**) Real time RT-PCR analysis for EPHA1, IRF6 and FADS1 expression was performed during TWIST driven EMT. EPHA1 and IRF6 were significantly decreased from day 2 and FADS1 was increased. (**B**) Heatmaps displaying all the probes for TRIM29, CDH1 and VIM, arranged in the genomic context.

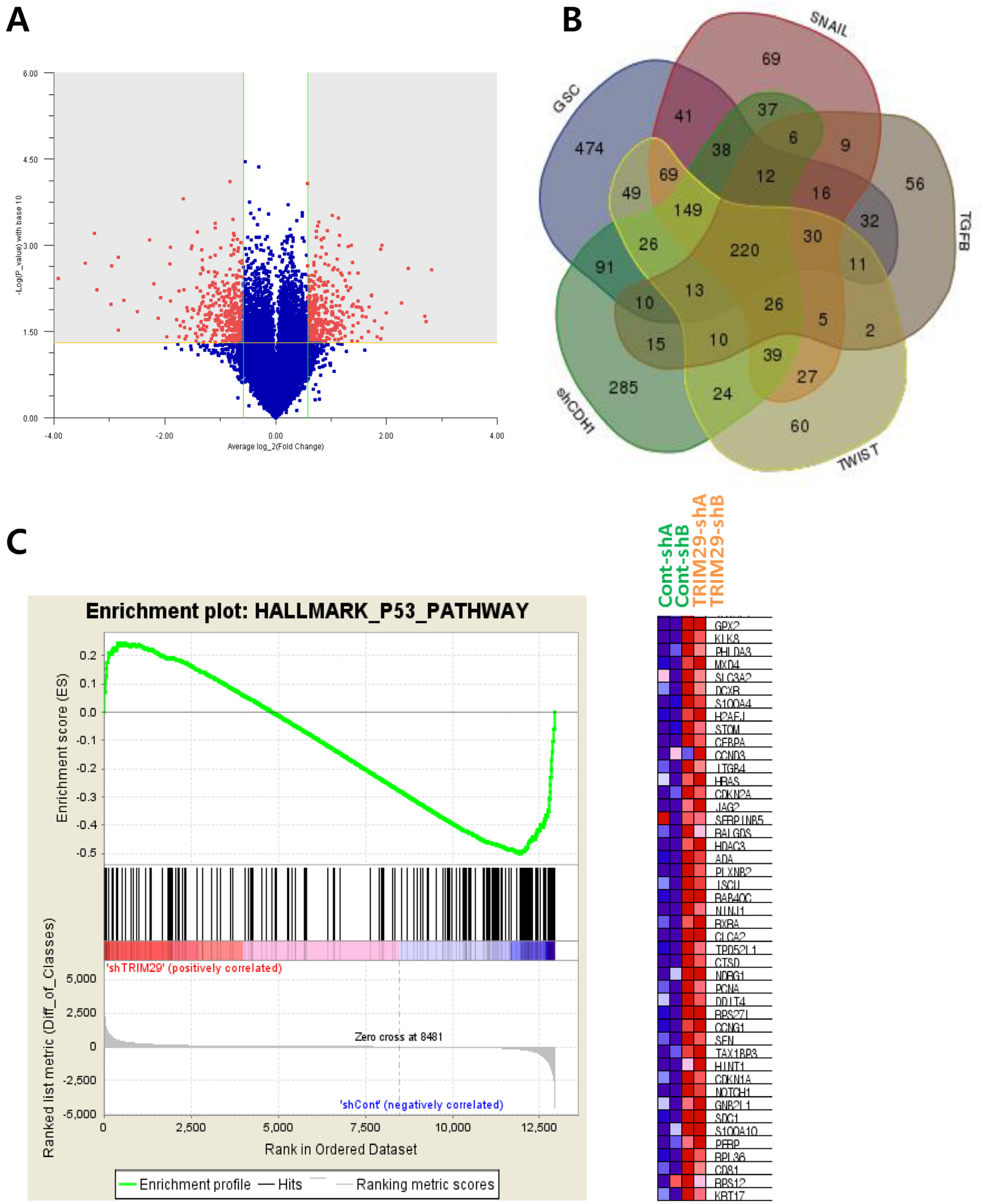

Supplementary Figure 4: (**A**) Volcano plots of gene expression differences for TRIM29 knockdown in HMEC (Significance Level 0.05, Fold Change Cutoff = 1.5, with Welch's *T*-Test). (**B**) Venn diagrams of the genes showing common genes in the core EMT signature. (**C**) GSEA of Hallmark_P53 pathway with TRIM29 downstream targets.

Supplementary Table 1: Genes of chromatin accessibility change during EMT. See Supplementary_ Table_1

Supplementary Table 2: Genes showing up and down regulation in the core EMT signature with TRIM29 knockdown

Common Up- regulated	Common Down-regulated
APOBEC3B	CA9
BTG1	CELSR2
CDH2	CLCA2
ECM2	FABP5
FADS1	FAT2
GNG11	FGFBP1
HRASLS3	FXYD3
IGFBP3	HSD17B8
LIMS1	JAG2
LPIN1	KLK10
PMP22	KLK7
RAPGEF2	KRT14
ROR1	KRT15
	KRT16
	KRT5
	MALL
	NMU
	PI3
	S100A7
	S100A8
	SDC1
	SERPINB13
	SERPINB3
	SFN
	SFRP1
	SLPI
	SNCA
	SPRR1A
	SPRR1B
	ST6GAL1
	TNFSF10
	TP73L
	TRIM29
	VSNL1
